# Leaf litter arthropod responses to tropical forest restoration

**DOI:** 10.1002/ece3.2220

**Published:** 2016-06-28

**Authors:** Rebecca J. Cole, Karen D. Holl, Rakan A. Zahawi, Philipp Wickey, Alan R. Townsend

**Affiliations:** ^1^ The Institute of Arctic and Alpine ResearchUniversity of ColoradoBoulderColorado80303; ^2^ Environmental Studies DepartmentUniversity of CaliforniaSanta CruzCalifornia95064; ^3^ Las Cruces Biological StationOrganization for Tropical StudiesApdo. 73‐8257San VitoCosta Rica; ^4^ Present address: University of Hawaii at ManoaHonoluluHawaii96822

**Keywords:** Applied nucleation, biodiversity, forest succession, litter fauna, macro‐arthropods, soil fauna, tropical forest restoration

## Abstract

Soil and litter arthropods represent a large proportion of tropical biodiversity and perform important ecosystem functions, but little is known about the efficacy of different tropical forest restoration strategies in facilitating their recovery in degraded habitats. We sampled arthropods in four 7‐ to 8‐year‐old restoration treatments and in nearby reference forests. Sampling was conducted during the wet and dry seasons using extractions from litter and pitfall samples. Restoration treatments were replicated in 50 × 50‐m plots in four former pasture sites in southern Costa Rica: *plantation* – trees planted throughout the plot; *applied nucleation/islands* – trees planted in patches of different sizes; and *natural regeneration* – no tree planting. Arthropod abundance, measures of richness and diversity, and a number of functional groups were greater in the island treatment than in natural regeneration or plantation treatments and, in many cases, were similar to reference forest. Litter and pitfall morphospecies and functional group composition in all three restoration treatments were significantly different than reference sites, but island and plantation treatments showed more recovery than natural regeneration. Abundance and functional group diversity showed a much greater degree of recovery than community composition. *Synthesis and applications*: The less resource‐intensive restoration strategy of planting tree islands was more effective than tree plantations in restoring arthropod abundance, richness, and functional diversity. None of the restoration strategies, however, resulted in similar community composition as reference forest after 8 years of recovery, highlighting the slow rate of recovery of arthropod communities after disturbance, and underscoring the importance of conservation of remnant forests in fragmented landscapes.

## Introduction

Tropical forests have been extensively cleared and altered over the past century with significant consequences for biodiversity and ecosystem function (Lamb 2014). An increased recognition of society's dependence on these forests for their services, such as carbon sequestration and hydrological cycling, has led to recent targets to restore hundreds of millions of hectares of forest globally (Replace with: (UN Climate Summit 2014), either through active means such as tree planting or passively via natural regeneration (Chazdon 2014; Lamb 2014). Tropical forest restoration has most commonly focused on re‐establishing native tree cover (Lamb 2011; Holl 2012) for a number of objectives, ranging from providing timber resources to restoring habitat for native flora and fauna. Restoration approaches, however, can result in markedly different community composition and habitat heterogeneity (Polley et al. 2005; Kattan et al. 2006; Holl et al. 2013). Moreover, the outcomes of restoration strategies, and their resulting differences in habitat structure, are poorly understood for many aspects of native biodiversity. This is especially true for soil and litter arthropod communities, despite the fact that they comprise an enormous proportion of tropical biodiversity (Decaens et al. 2006; Hamilton et al. 2013) and drive critical underlying ecosystem processes (Lavelle 1996).

The most common approach for actively restoring deforested lands in the tropics is to establish tree plantations over large areas (reviewed in Lamb 2011; Holl 2012). This strategy, however, can result in relatively homogeneous conditions in terms of tree species diversity, and habitat structure (Corbin and Holl 2012) may leave a strong legacy effect of the planted tree species on nutrient cycling and community composition of recruiting species (Cusack and Montagnini 2004; Boley et al. 2009) and is resource intensive. Applied nucleation (also called “woodland islets” or “cluster planting”) is a low‐cost active restoration technique that simulates natural forest succession by establishing small patches or “islands” of trees that spread over time through tree growth and by facilitating recruitment at patch edges (Yarranton and Morrison 1974; Rey Benayas et al. 2008; Corbin and Holl 2012). Past research at the current study location has shown that applied nucleation results in higher variability of tree canopy openness than either mixed‐species plantations or sites regenerating naturally (i.e., passive regeneration; Holl et al. 2013). Moreover, there is a stronger effect on nutrient cycling in the plantation treatment, where planted trees produce abundant litter that is higher in N but lower in Ca, Mg, and K than nearby secondary forest (Celentano et al. 2011b). These differences in structural variability and leaf litter chemistry may lead to differential effects on leaf litter arthropods, which are the focus of the current study.

Soil communities, including organisms that decay plant material and leaf litter, are thought to be among the most taxonomically diverse on earth (Anderson 1975; Wolters 2001; Decaens et al. 2006). Tropical forest soil communities are particularly species rich due to a high degree of resource and microhabitat heterogeneity (Coleman 2001; Bardgett 2002), where arthropod communities perform a variety of important roles in ecosystem functioning. For example, saprophagous arthropods (detritivores) such as millipedes (Diplopoda), woodlice (Isopoda), and earwigs (Dermaptera) are litter transformers in terrestrial ecosystems (Wardle et al. 2002), whereas ecosystem engineers such as termites (Isoptera) and ants (Formicidae) can affect patterns of soil formation (Jouquet et al. 2006). Arthropod diversity and density play key roles in rates of litter decomposition (Lavelle 1996; Gonzalez and Seastedt 2001; Wardle et al. 2002) and nitrogen mineralization (Anderson and Ineson 1984; Carcamo et al. 2000; Hattenschwiler and Bretscher 2001; David 2014), factors that may, in turn, affect the plant community structure (De Deyn et al. 2004). Soil arthropods also influence soil structure and porosity, which can affect hydrological processes (Lavelle 1996).

Despite the importance of soil and litter arthropods in ecosystem function, only a few studies have examined how these communities respond to different restoration strategies that target tropical forest recovery. Studies in single‐species tree plantations have shown that the tree species planted can impact arthropod abundance (Kattan et al. 2006) and that arthropod diversity and community composition are correlated with litter chemistry, particularly N, P, and C:N ratios in plantations (Warren and Zou 2002) and intact forests (Sayer et al. 2010; Ashford et al. 2013). Studies focusing on specific faunal groups such as beetles (Gardner‐Gee et al. 2015) and ants (Cristescu et al. 2012) have shown that diversity and abundance are greater in planted native forests and reference forests compared to highly disturbed areas such as exotic pastures and mine sites. Research on patterns of arthropod diversity in agricultural landscapes (Perfecto et al. 1997; Philpott et al. 2006), home gardens (Lemessa et al. 2015), and reference forest canopies (Basset et al. 2012) shows a relatively strong association with plant species diversity and habitat heterogeneity. Accordingly, restoration approaches that create more heterogeneous habitat and more closely approximate intact forest should result in greater arthropod species diversity (Weibull et al. 2003; Schmidt and Tscharntke 2005). But, the extent to which different restoration approaches lead to recovery of forest arthropod communities is largely unknown.

In this study, we compare leaf litter arthropod communities under three restoration strategies (natural regeneration, applied nucleation/tree islands, and mixed‐species tree plantations) and adjacent reference forests to evaluate how effective the different restoration approaches are in restoring arthropod abundance, richness, and community composition relative to reference forest ecosystems.

## Materials and Methods

### Study region

The study was carried out in four experimental forest restoration sites and two reference forest sites in a mixed‐use agricultural landscape between the Las Cruces Biological Station (LCBS; 8°47′7″N; 82°57′32″W) and Agua Buena (8°44′42″N; 82°56′53″W) in southern Costa Rica. The forest in this region is classified as tropical premontane rain forest (Holdridge et al. 1971). Study sites range in elevation from 1110 to 1180 m a.s.l. and receive 3500–4000 mm annual rainfall with a distinct dry season from December to March. Reference forest fragments and restoration sites are surrounded by a mosaic of mixed‐use agricultural land and small forest remnants (Zahawi et al. 2015).

### Experimental design and site characteristics

We sampled leaf litter arthropods in four habitats: reference forests and three restoration treatments (plantation, applied nucleation – hereafter “island,” and natural regeneration). The restoration treatments were established in ~1‐ha blocks with each treatment in a 50 × 50‐m plot (Figure S1). Each block was cleared by machete prior to the establishment of the restoration treatments (2004–2005) in order to have similar initial conditions (Holl et al. 2011). The plantation and island treatments were planted with a mix of two native timber species, *Terminalia amazonia* (J.F. Gmel.) Exell (Combretaceae) and *Vochysia guatemalensis* Donn. Sm. (Vochysiaceae), and two naturalized N‐fixing legume species, *Erythrina poeppigiana* (Walp.) O.F. Cook and *Inga edulis* Mart. Plantations were uniformly planted with trees separated by 2.8 m; island treatments consisted of six planted tree islands of three sizes: two each of 4 × 4, 8 × 8, and 12 × 12 m (Figure S1). Tree species, planting density, and species composition were identical in planted areas. Trees were planted in ~20% of the area of island treatments, but by 2012, islands had expanded through tree growth and new recruitment to cover 52.5 ± 14.8% (SD) of the total 50 × 50‐m area (K. D. Holl and R. A. Zahawi, unpubl. data). The natural regeneration treatments were allowed to undergo passive recovery (i.e., no tree planting). The estimated cost of the different planting treatments was $1461–1281 ha^−1^ for plantations and $401–626 ha^−1^ for island treatments based on the cost of planting and maintaining seedlings in the first 2.5 years (Zahawi and Holl 2009). The four sites used in this study all had well‐established restoration treatments (Table S1; Holl et al. 2011) and are separated by >2.5 km.

Two reference forest plots were located in each of the two largest and most intact remnant forests in the region, including LCBS and Finca Loma Linda (330 and 48 ha; *n* = 4 plots total); plots within each forest were separated by ≥400 m. The reference forests used in this study have highly diverse canopies with over 300 native tree species (LCBS 2014) and relatively intact understories.

Tree canopy cover was measured in each restoration plot in June–July 2011 (1 year prior to arthropod data collection) using a densiometer (Holl et al. 2013). Canopy cover was nearly continuous in reference forest and plantations, whereas natural regeneration plots had a sparse canopy comprised of a mix of ruderal shrubs and isolated small trees. Average canopy cover in the island treatments was intermediate and more heterogeneous (i.e., had a higher coefficient of variation), as tree island canopy and understory cover the resembled plantations, whereas the vegetation between tree islands was more similar to natural regeneration plots (Holl et al. 2013). Reference forest understories were dominated by native plants. Understories of the plantations had minimal exotic forage grass cover with interspersed, naturally recruited native trees and shrubs (K. D. Holl and R. A. Zahawi, unpubl. data). Ground cover in plantations and the interiors of planted tree islands had a thick layer of leaf litter dominated by *I. edulis* leaves (Celentano et al. 2011a), whereas leaf litter in reference forests comprised of a diversity of species. Natural regeneration plots and unplanted areas of island plots were dominated by the exotic forage grasses that had initially covered all restoration sites: primarily *Axonopus scoparius* (Flugge) Kuhlm. and *Urochloa brizantha* (Hochst. Ex. A. Rich.) R.D. Webster, the vine *Heterocondylus vitalbae (D.C.)* King & H. Robins, the fern *Pteridium arachnoideum* (Kaulf.) Maxon, and ruderal shrubs, such as *Vernonanthura patens* (Kunth) H. Rob. and *Vernonia arborescens* (L.) Sw.

### Arthropod sampling

We collected ground‐dwelling macro‐arthropods (visible without magnification, >1 mm) in litter over 2‐week periods in March (dry season) and August (wet season) 2012, 7–8 years after the initiation of the study. Litter was collected from nine sampling points systematically distributed throughout the 50 × 50‐m area in each plantation, natural regeneration, and reference forest plot (Figure S1). In island treatments, we collected three litter samples from inside the planted tree patches and six samples from the unplanted areas ≥8 m away from the tree patches (Figure S1) to represent both “habitats.” At each sampling point, we placed a 50 × 50‐cm metal frame on the ground and quickly moved all litter inside the frame into a cloth‐collecting bag. The 9 litter samples from each plot were pooled and sieved through a 2‐cm mesh and placed in Winkler extractors for 48 h. We inspected the litter remaining after sifting and the litter left in the Winkler extractors by hand to collect any remaining visible fauna. In March 2012, we also placed one pitfall trap 3 m from each litter sampling location for 48 h. Pitfall traps consisted of 8‐cm‐diameter plastic cups half filled with ethanol and water. The nine pitfall samples from each plot were pooled. All individuals collected were quantified under a stereoscopic microscope. Mature insects were identified by A. Solis (Instituto Nacional de Biodiversidad, Costa Rica) to at least family level and to genus and species when possible. Individuals that could not be identified to species level were assigned a morphospecies. Noninsect arthropods were identified only to order the level following McGavin (2000). All taxa were classified by functional groups. Arthropods, most frequently Formicidae, with the known mixed foraging strategies, or for which the dominant functional group could not be determined, were placed in a “mixed” category that included predators, detritivores, and nectarivores.

### Soil nutrients

In July 2012, we collected 25, 2.5‐cm‐diameter × 15‐cm‐deep soil cores in each of the 12 restoration plots (4 sites × 3 treatments) and in two of the reference forest plots, one in each fragment. The samples from each plot were bulked and homogenized, air‐dried, and passed through a 2‐mm sieve. Samples were analyzed for total C, N, K, and C:N ratios, Mehlich III P, and other major cations following the standard procedures at Brookside Laboratories, New Bremen, OH (see www.blinc.com/worksheet_pdf/SoilMethodologies.pdf and Gavlak et al. (2003) for details on protocols).

### Statistical analyses

Arthropod abundances from litter were standardized to number of individuals per m^2^, and those collected using pitfall traps were standardized to individuals per trap. Total abundance and abundances by orders and functional groups were calculated separately for litter and pitfall samples. We used several measures of richness and diversity including the number of morphospecies, orders, and functional groups. We also estimated morphospecies individual sample‐based richness (Chao 1 from Chao 1987) using EstimateS (Colwell 2013) and calculated Simpson's diversity index, *D* = [Σ*n*_*i*_(*n*_*i*_ − 1)]/[*N*(*N* − 1)], where *n*_*i*_ and *N* are the number of individuals and total individuals in each plot.

Preliminary analyses showed that litter arthropod morphospecies richness and abundance were similar across collections in dry season (62.1 morphospecies and 47.8% of total abundance) and wet season (65.4 morphospecies and 52.2% of total abundance), as were differences among treatments; therefore, we combined these data for all further analyses. Total abundance and taxon abundance within orders and functional groups, all measures of richness and diversity, soil variables, and canopy height and openness (restoration plots only) were compared across the habitat types using either one‐way ANOVA followed by Tukey's multiple comparison test when habitat type was significant or Kruskal–Wallis nonparametric ANOVA when data did not meet assumptions of parametric statistics. In some cases, data were transformed to meet assumptions of normality and homogeneity of variances. We regressed total abundance and the number of morphospecies in the restoration treatments against soil percent C, N, P, K, and C:N ratios. We calculated Pearson's correlation coefficients between the coefficient of canopy openness at the plot level (using data from Holl et al. 2013) and measures of richness. All univariate analyses were performed using Systat 13 (Systat Software, Chicago, IL). We consider α = 0.05 as significant and report means ± 1 SE throughout.

Differences in arthropod community composition among the four habitat types were visualized with nonmetric multidimensional scaling (NMS) followed by permutational multivariate analysis of variance (PERMANOVA) based on Bray–Curtis dissimilarity. Preliminary analyses of litter and pitfall samples based on morphospecies presence/absence data (to account for the behavior of social insects: e.g., Longino et al. 2002) and based on morphospecies abundance showed identical trends. Because abundance data are more informative, the results of the abundance‐based multivariate analyses are presented here. Differences in functional group structure among the four habitat types were compared with NMS and PERMANOVA based on Bray–Curtis dissimilarity of litter and pitfall abundance data separately. Multivariate analyses were performed using PCORD 5.31 (MjM Software Design, Gleneden Beach, OR) and PAST 3.05 statistical software (Hammer et al. 2001).

## Results

We identified a total of 4753 arthropod individuals (3095 collected from litter samples and 1658 from pitfall traps) representing 25 orders, 52 families, and 302 species/morphospecies. The orders with the greatest numbers of individuals were Hymenoptera (litter 23.0%, pitfall 52.5%) and Coleoptera (litter 21.1%, pitfall 12.3%; Table S2). The most abundant functional groups were detritivores (27.9%) and predators (24.4%) for litter samples, and the mixed category (52.5%, primarily omnivores), and detritivores (18.2%) in pitfall samples (Table S2).

**Figure 1:**
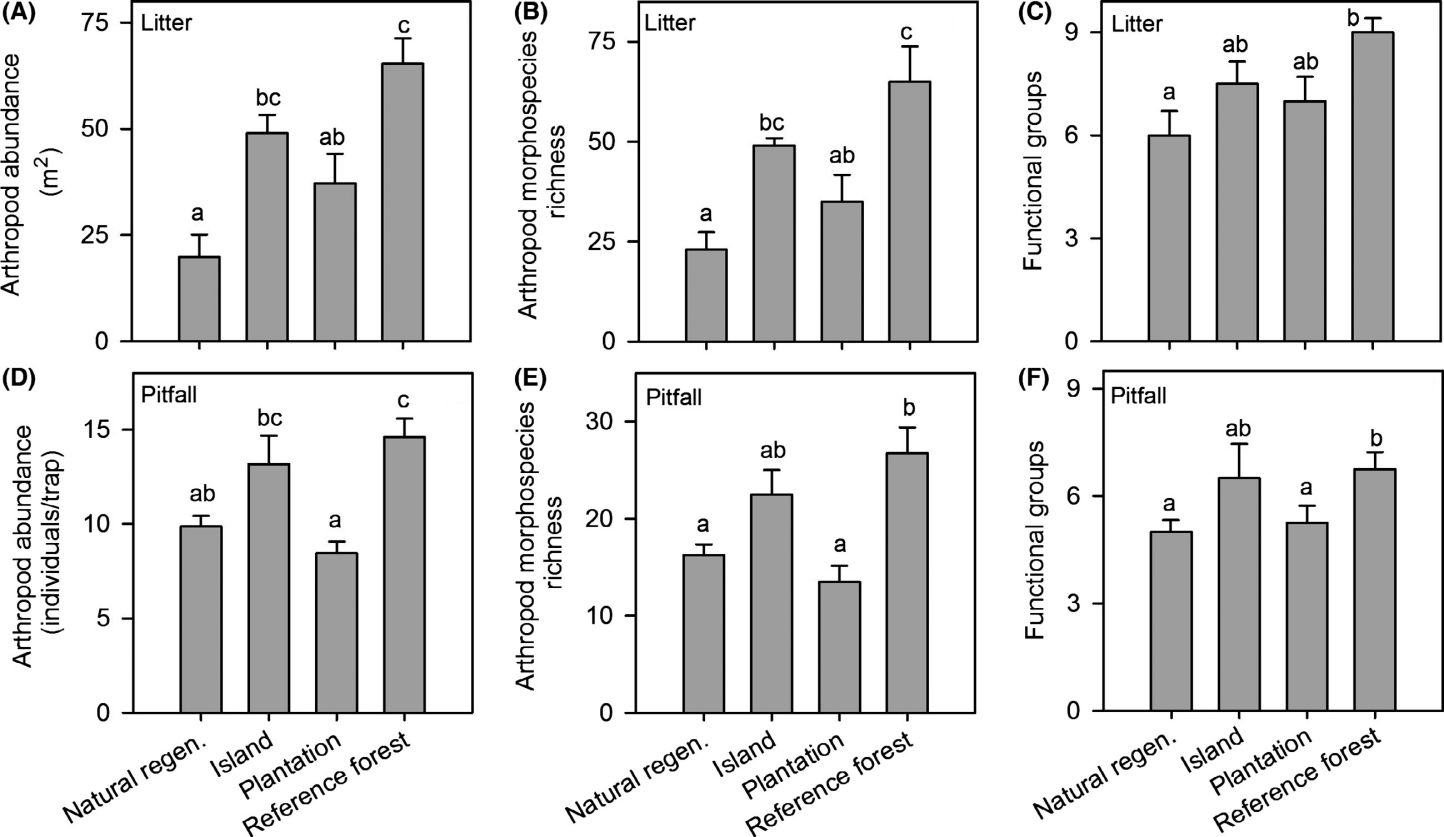
Litter and pitfall sample arthropod abundance (A, D), morphospecies richness (B, E), and the number of functional groups (C, F) in four habitat types. Error bars indicate +1 SE. Means with the same letter are not significantly different using Tukey's multiple comparison procedure (*P* < 0.05).

Abundance was lowest in natural regeneration and plantation plots, and highest in reference forest (litter *F*_3,12_ = 11.3, *P* < 0.001, pitfall, *F*_3,12_ = 8.2, *P* < 0.003, Fig. 1A and D). Among restoration treatments, island plots had the highest arthropod abundances for both litter and pitfall samples (Fig. 1A and D) and did not differ statistically from reference forests. Overall differences in abundance were largely driven by Coleoptera, Hymenoptera, and Araneae in litter samples and Coleoptera in pitfall samples (Table S2). Of the functional groups in litter samples, detritivores, including Blattaria, the family Curculionidae (order Coleoptera), Dermatera, and Diplopoda, were less abundant in natural regeneration than the other three habitat types (Table S3). Predators, primarily the families Carabidae and Staphylinidae (order Coleoptera) and Araneae, were much more abundant in litter samples from reference forests and island treatments. In pitfall samples, detritivores, mainly the family Nitidulidae (order Coleoptera), were most abundant in reference forests, whereas fungal‐feeding arthropods in the families Mycetophagidae and Ptiliidae (order Coleoptera) were found most often in islands and reference forests (Table S3).

Reference forest had the greatest number of arthropod morphospecies, and natural regeneration and plantation treatments had the lowest, with intermediate values in the island treatment (litter *F*_3,12_ = 9.0, *P* = 0.002, pitfall, *F*_3,12_ = 8.3, *P* = 0.003, Fig. 1B and E). This was also the trend for number of functional groups (litter *F*_3,12_ = 3.9, *P* = 0.036, pitfall, *F*_3,12_ = 4.3, *P* = 0.027, Fig. 1C and F). Interestingly, Chao 1 morphospecies richness values for both litter and pitfall samples from islands were as high as reference forests. Diversity (Simpson's index) was significantly lower in natural regeneration compared to both the reference forest and islands for pitfall samples, but did not differ among habitat types for litter samples (Table 1).

**Table 1:** Measures of arthropod morphospecies richness and diversity in four habitat types compared using one‐way ANOVA. Values are means ± 1 SE

Measure	Habitat type				*F*	*P*
	Natural regeneration	Island	Plantation	Reference forest		
Leaf litter						
Chao 1 richness	32.7 ± 8.0^a^	81.7 ± 6.7^b^	42.0 ± 8.4^a^	79.4 ± 12.7^b^	7.5	0.004
Simpson's diversity index (1 − D)	0.9 ± 0.1	0.9 ± 0.1	0.9 ± 0.1	0.9 ± 0.1	1.3	0.309
Order richness	11.0 ± 1.3^a^	14.5 ± 1.0^ab^	13.5 ± 1.0^ab^	15.5 ± 0.9^b^	3.9	0.036
Pitfall						
Chao 1 richness	36.6 ± 6.7^ab^	57.1 ± 12.1^b^	18.3 ± 3.6^a^	51.8 ± 4.4^b^	8.2	0.003
Simpson's diversity index (1 − D)	0.8 ± 0.1^a^	0.9 ± 0.1^b^	0.9 ± 0.1^ab^	0.9 ± 0.0^b^	4.8	0.021
Order richness	7.8 ± 0.9^ab^	8.8 ± 0.8^b^	5.3 ± 0.3^a^	9.3 ± 0.5^b^	9.3	0.002

Means with the same letter are not significantly different using Tukey's multiple comparison procedure (*P* < 0.05).

Surface soil C, N, P, K, and C:N ratios were similar across the three restoration treatments, but *K* values were higher in the two reference forest sites where soil samples were collected (Table 2). Mean canopy cover was highest in plantations (11.1 ± 0.4), intermediate in island treatments (7.0 ± 1.1), and lowest in natural regeneration (4.8 7.0 ± 1.4; *F*_2,9_ = 20.8, *P* = 0.001). The coefficient of variation for canopy openness, however, was significantly greater (*F*_2,9_ = 9.2, *P* = 0.006) in island treatments (60.7 ± 7.6) than in natural regeneration (32.4 ± 8.7) or plantations (20.1 ± 5.5). There were no significant relationships between arthropod abundance or diversity/richness measures and soil variables (*r* ^2^ < 0.5, *P* > 0.05 in all cases). Chao 1 morphospecies richness was correlated with the coefficient of variation of canopy openness in restoration plots (litter, *r* = 0.74, *P* = 0.006, pitfall, *r* = 0.68, *P* = 0.016, *n* = 12), whereas absolute morphospecies richness was only significantly correlated in pitfall traps (litter, *r* = 0.47, *P* = 0.120, pitfall, *r* = 0.64, *P* = 0.026, *n* = 12).

**Table 2:** Soil variables in four habitat types compared using one‐way ANOVA. Values are means ± 1 SE

Soil variable	Habitat type				*F*	*P*
	Natural regeneration	Island	Plantation	Reference forest		
C (%)	7.6 ± 0.9	6.6 ± 1.0	7.5 ± 1.6	7.4 ± 2.5	0.2	0.926
N (%)	0.6 ± 0.1	0.5 ± 0.1	0.6 ± 0.1	0.6 ± 0.2	0.9	0.920
C:N	12.7 ± 0.4	12.1 ± 0.4	11.9 ± 0.5	12.0 ± 0.4	0.8	0.532
Mehlich P (mg·kg^−1^)	4.0 ± 0.1	3.3 ± 1.3	2.8 ± 0.3	7.0 ± 1.0	3.4	0.060
K (mg·kg^−1^)	135.0 ± 34.3^a^	109.0 ± 29.5^a^	66.8 ± 3.5^a^	258.5 ± 37.5^b^	8.3	0.005

Means with the same letter are not significantly different using Tukey's multiple comparison procedure (*P* < 0.05).

**Figure 2:**
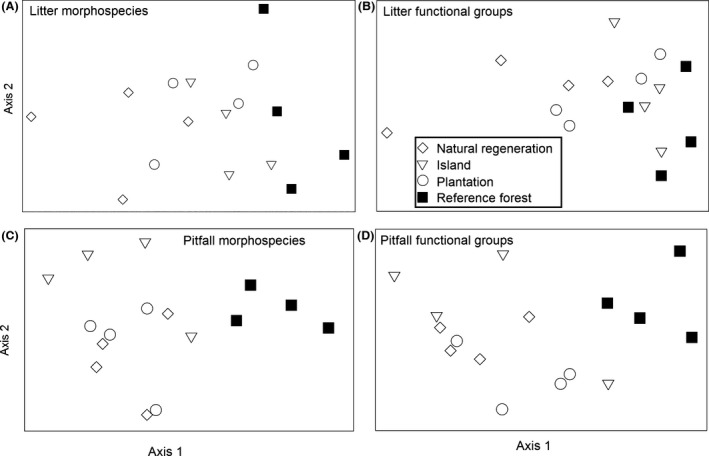
Nonmetric multidimensional scaling plots of arthropod community composition and functional groups in the four habitat types. Bray–Curtis was used as a similarity measure. (A) Litter morphospecies abundance (stress = 0.1315); (B) litter functional group abundance (stress = 0.065); (C) pitfall morphospecies abundance (stress = 0.1631); (D) pitfall function group abundance (stress = 0.094).

Nonmetric multidimensional scaling and PERMANOVA of morphospecies abundance showed significant treatment differences in community composition for both litter (*F*_3,12_ = 2.5, *P* = 0.001) and pitfall samples (*F*_3,12_ = 3.3, *P* = 0.001). For the litter samples, reference forest differed from all the restoration treatments, while plantations and islands were similar to each other, but differed from natural regeneration (*P* < 0.05 in all comparisons) (Fig. 2; Table S4). For pitfall samples, reference forests were similar to plantations and islands, but differed from natural regeneration; islands differed from natural regeneration (Fig. 2; Table S4). Functional group composition in reference forest litter samples differed significantly from natural regeneration plots (*F*_3,12_ = 3.7, *P* = 0.001), but was similar to plantations and islands. Functional group composition of reference forest pitfall samples differed from all of the restoration treatments (*F*_3,12_ = 4.0, *P* = 0.001) (Fig. 2; Table S4).

## Discussion

### Arthropod abundance, richness, and diversity

Overall, there was greater abundance, richness, and diversity in the applied nucleation/islands compared to the other restoration treatments, while reference forests frequently had the highest values. For example, the applied nucleation/island treatments hosted the greatest number of arthropods of the three restoration strategies and were similar in this respect to the reference forest. The nucleation/island treatments also tended to have higher overall number of orders, morphospecies, and functional groups when compared to natural regeneration and were similar to reference forest. By contrast, the most intensive restoration approach, tree plantations, had low total measures of abundances and richness and did not differ from natural regeneration.

These results are surprising given that our prior research shows that plantations have much higher litter biomass than natural regeneration (Celentano et al. 2011b), a factor that has often been associated with greater arthropod abundance (Wardle et al. 2002; Kaspari and Yanoviak 2008; Sayer et al. 2010; but see Donoso et al. 2010). Leaf litter chemistry can also affect arthropod community composition and abundance (Hattenschwiler and Gasser 2005; Kattan et al. 2006), and the higher abundance and diversity of arthropods in island treatments may reflect greater litter chemical diversity. The litter in plantations was strongly dominated (>70%) by *I. edulis* (Celentano et al. 2011b), whereas litter in the island treatments was a mix of planted trees and natural regeneration. *Inga edulis* litter is higher in N, but lower in Ca, Mg, and K than the other restoration treatments. It is also slower to decompose than the litter in secondary forests, which comprises of leaves from many species (Celentano et al. 2011a, 2011b). Surprisingly, we did not find correlations between soil variables and measures of arthropod abundance and richness, despite the fact that other studies have shown these to be important drivers of arthropod community structure (McGlynn et al. 2007; Sayer et al. 2010; Ashford et al. 2013). That said, it is possible that soil measurements were not carried out at a fine enough scale spatially or temporally to capture differences across restoration treatments.

The trend for higher arthropod abundance and richness in island as compared to other restoration treatments could be also influenced by greater variability in canopy openness in these plots (Holl et al. 2013), which can affect light availability, throughfall, and soil moisture. These factors influence biotic conditions, resource availability, and arthropod communities in tropical forests (Jansen 1997; Richardson et al. 2010) and are consistent with prior studies showing that greater structural complexity of habitats provides more niches and diverse ways for exploiting environmental resources, thereby increasing species diversity (e.g., Tews et al. 2004). This notion is supported by other surveys of arthropods in transformed tropical landscapes in Colombia (Cabra‐Garcia et al. 2012), agricultural landscapes in Ethiopia (Lemessa et al. 2015), and forestry plantations in Puerto Rico (Warren and Zou 2002), all of which found to be strong associations between habitat heterogeneity and greater arthropod richness.

High arthropod abundance in the reference forest was expected and is consistent with the few prior studies on the effects of tropical forest restoration on litter arthropods. For example, surveys of litter‐dwelling ants in tropical and subtropical Australia reported that rainforests had the highest abundances, while reforested sites were intermediate and abandoned pastures were lowest (Piper et al. 2009). The comparable levels of abundance and diversity between reference forests and island treatments in the current study are certainly promising, although not necessarily an indicator that community structure has recovered as discussed below.

### Composition and functional groups

Community composition, based on NMDS of morphospecies, differed substantially across the four habitat types with the reference forest being distinctly different from all restoration treatments. Among the restoration treatments, applied nucleation/islands and plantations were more similar to each other than to natural regeneration plots. A similar pattern emerged in the functional group composition. The greatest difference in both litter and pitfall functional group compositions was between the reference forest and the natural regeneration treatment. Functional group composition differences among the restoration treatments varied somewhat based on collection method, which likely reflects the different capture efficiencies of the two methods. Litter samples are widely considered to be a useful quantitative collection method with more even representation across arthropod groups, whereas pitfall traps are more qualitative, capturing a greater diversity of surface‐active arthropods across the landscape (e.g., Topping and Sunderland 1992; Oliver and Beattie 1996; Work et al. 2002; Sabu and Shiju 2010; Silva et al. 2013). Taking this into account, the collective results of morphospecies and function group composition point to a strong trend of recovery in both the island and plantation treatments compared to natural regeneration.

The patterns in functional diversity are consistent with differences in structure and resources across the habitats. For example, detritivores were the least abundant in the natural regeneration plots, which are consistent with expectations based on the low litter mass in these treatments (Celentano et al. 2011a). Predators, namely arachnids (Araneae) and predatory Coleoptera, drove patterns of high arthropod abundance in the island treatments and reference forests. These patterns likely reflect more variable litter, vegetation, and food web structure (e.g., Jacquemin et al. 2012). Reference forests hosted significantly higher abundances of mycophagous arthropods, presumably due to the greater presence of fungi and belowground fungal mutualisms that are linked to aboveground insect diversity (e.g., Bennett 2010).

The few studies that have assessed arthropod functional groups across tropical forests have also recorded responses to habitat degradation. For example, Burghouts et al. (1992) found declines in predators (Pseudoscorpiones) and detritivores (Isoptera) following logging in Malaysia. In forests in Borneo, Edwards et al. (2012) determined that the abundance of litter predators, omnivores, and detritivores was lower in naturally regenerating logged forests compared to primary forests although there were no differences in herbivore abundances.

Arthropod abundance and functional diversity appear to be recovering more quickly than community composition, a trend consistent with other recent tropical secondary forest studies (Ottermanns et al. 2011; Curran et al. 2014). Tropical forest arthropod diversity measures are partly based on assumed specializations with plant species (e.g., Erwin 1982), and the greater tree species diversity in primary forests likely sustains higher levels of arthropod diversity (May 1988; Novotny and Basset 2005; Novotny et al. 2006). In addition, the broad range of microclimates and high biogeochemical heterogeneity within tropical forests (Townsend et al. 2008) can affect resources important to arthropod dynamics. That said, although studies of forest canopies have shown strong relationships between arthropod diversity and tree diversity (Basset et al. 2012), specialization in litter communities may be lower because many organisms are considered generalists (Donoso et al. 2010). The high levels of abundance, richness, and diversity we found in island plots may be due to these treatments having species characteristic of both open areas (natural regeneration plots) and planted canopy cover (like plantations), rather than their hosting more mature forest species. It is also possible that arthropod communities under these different restoration techniques may follow divergent recovery trajectories. One of the few studies of litter arthropod communities in restored habitat found that community composition differed among remnant sclerophyll forests, rehabilitated mine sites, and pasture land in Australia (Nakamura et al. 2003). However, factors limiting the recolonization and recovery of soil and litter arthropod communities in successional habitats remain largely unexplored.

### Management recommendations

Taken together, our results suggest that restoration approaches that establish greater habitat heterogeneity, such as applied nucleation, can lead to similar or higher levels of abundance, richness, and diversity for some components of the native soil and litter arthropod fauna. Given that this restoration approach also is less costly and similarly effective in facilitating forest seedling recruitment as the more intensive plantation restoration approach (Holl et al. 2011; Zahawi et al. 2013), we consider it to be a strategic method. That said, arthropod community composition in all the restoration methods was distinct from reference forests at this stage in recovery, which is not surprising given that recent meta‐analyses indicate that full recovery of a range of measures in tropical forests requires several decades to a century (Jones and Schmitz 2009; Rey Benayas et al. 2009; Curran et al. 2014). The trend toward recovery of functional diversity and functional group composition in restoration is promising, however, and suggests that at least some ecosystem functions may recover ahead of species composition. This study underscores the often unpredictable nature of recovery and highlights the importance of protecting forest remnants to conserve a region's full complement of biodiversity.

## Conflict of Interest

None declared.

## Supporting information


**Table S1.** Elevation and site use history.
**Table S2.** Abundance within orders for litter samples (individuals m^−2^) and pitfall samples (individuals/trap) in each habitat type (*N* = 4).
**Table S3.** Arthropod functional diversity density per m^2^ for litter samples and per trap for pitfall samples in four habitat types.
**Table S4.** PERMANOVA analysis results for litter and pitfall morphospecies and functional group data.
**Figure S1.** Approximate arthropod sampling locations (●) in each of the four habitat types.
