# Supplementary material for: Leaf litter arthropod responses to tropical forest restoration
Source: Ecol Evol. 2016 Jun 28;6(15):5158–68. doi: 10.1002/ece3.2220 (PMC4984494; doi:10.1002/ece3.2220)

Supplementary Tables and Figures

| **Supp. Table 1.** Elevation and site use history. | | | |
| --- | --- | --- | --- |
| **Site** | **Type** | **Elevation** | **Site use history** |
| LL1 | Reference forest | 1160 | Primary forest (330 ha) |
| LL2 | Reference forest | 1175 | Primary forest (330 ha) |
| LCBS1 | Reference forest | 1150 | Primary forest (48 ha) |
| LCBS2 | Reference forest | 1160 | Primary forest (48 ha) |
| HB | Restoration | 1120 | Coffee (25 yr), pasture (8 yr), fallow (4 yr) |
| JG | Restoration | 1180 | Mixed simultaneous uses: mostly beans (35 yr) and fallow (5 yr), partly coffee (30 yr) and pasture (10 yr) |
| OM | Restoration | 1120 | Beans and corn (10 yr), pasture (5 yr), coffee (5 yr), fallow (5 yr) |
| SG | Restoration | 1110 | Coffee (25 yr), fallow (3 yr), pasture (4 yr) |
| *Land-uses are based on land owner interviews (Holl et al 2011).* | | | |

| **Supp. Table 2.** Abundance within orders for litter samples (individuals/m^2^) and pitfall samples (individuals/trap) in each habitat type (N=4). Values are means ± 1 SE. Variables were compared using ANOVA, unless noted with superscript K indicating a nonparametric Kruskal-Wallis analysis | | | | | | | |
| --- | --- | --- | --- | --- | --- | --- | --- |
| **Taxon** | | **Natural regeneration** | **Island** | **Plantation** | **Reference forest** | **F/KS test statistic** | **P** |
| **Litter samples** | |  |  |  |  |  |  |
| *Arachnida* | |  |  |  |  |  |  |
|  | Acarina | 0.5 ± 0.3 | 1.3 ± 0.4 | 0.7 ± 0.3 | 1.4 ± 0.6 | 1.6 | 0.237 |
|  | Araneae | 1.9 ± 0.4 | 4.9 ± 1.2 | 2.8 ± 0.5 | 6.6 ± 2.0 | 2.5 | 0.111 |
|  | Opiliones | 0.3 ± 0.2^a^ | 0.8 ± 0.1^ab^ | 1.1 ± 0.2^b^ | 0.3 ± 0.1^ab^ | 3.8 | 0.041 |
|  | Pseudoescorpiones | 0.1 ± 0.1 | 0.3 ± 0.2 | 0.7 ± 0.2 | 0.2 ± 0.1 | 3.0^K^ | 0.392 |
| *Annelida* | | 0.0 ± 0.0 | 0.6 ± 0.3 | 0.4 ± 0.1 | 0.1 ± 0.1 | 6.6^K^ | 0.085 |
| *Crustacea* | |  |  |  |  |  |  |
|  | Isopoda | 1.9 ± 1.0 | 2.9 ± 1.6 | 2.3 ± 1.2 | 3.8 ± 1.3 | 0.4 | 0.736 |
| *Entognatha* | |  |  |  |  |  |  |
|  | Collembola | 0.3 ± 0.3 | 0.4 ± 0.2 | 0.4 ± 0.4 | 1.2 ± 0.4 | 4.8^K^ | 0.189 |
| *Insecta* | |  |  |  |  |  |  |
|  | Blattaria | 0.7 ± 0.3 | 2.0 ± 0.3 | 3.4 ± 0.9 | 4.0 ± 1.2 | 5.4 | 0.140 |
|  | Coleoptera | 2.7 ± 0.2^a^ | 7.8 ± 0.7^bc^ | 5.6 ± 1.0^b^ | 13.9 ± 3.6^c^ | 9.3^K^ | 0.029 |
|  | Dermaptera | 0.0 ± 0.0^a^ | 0.9 ± 0.3^ab^ | 2.2 ± 1.4^bc^ | 3.1 ± 0.7^c^ | 8.5^K^ | 0.036 |
|  | Diplura | 0.3 ± 0.3 | 0.6 ± 0.6 | 0.1 ± 0.1 | 0.1 ± 0.1 | 0.8^K^ | 0.858 |
|  | Diptera | 0.0 ± 0.0 | 0.0 ± 0.0 | 0.0 ± 0.0 | 0.1 ± 0.1 | - | - |
|  | Hemiptera | 0.9 ± 0.6^a^ | 2.1 ± 0.1^ab^ | 0.7 ± 0.4^a^ | 3.2 ± 0.6^b^ | 5.2 | 0.015 |
|  | Homoptera | 0.0 ± 0.0 | 0.0 ± 0.0 | 0.1 ± 0.1 | 0.0 ± 0.0 | - | - |
|  | Hymenoptera | 5.4 ± 2.3 | 12.2 ± 1.0 | 5.9 ± 1.9 | 9.3 ± 1.3 | 2.5 | 0.114 |
|  | Isoptera | 0.0 ± 0.0 | 0.7 ± 0.7 | 0.2 ± 0.2 | 0.0 ± 0.0 | 0.4 | 0.736 |
|  | Orthoptera | 0.2 ± 0.2^a^ | 0.6 ± 0.2^ab^ | 0.2 ± 0.2^a^ | 1.1 ± 0.3^b^ | 8.1^K^ | 0.044 |
|  | Phasmatodea | 0.2 ± 0.2 | 0.0 ± 0.0 | 0.0 ± 0.0 | 0.0 ± 0.0 | - | - |
|  | Siphonaptera | 0.0 ± 0.0 | 0.0 ± 0.0 | 0.0 ± 0.0 | 0.1 ± 0.1 | 4.8^K^ | 0.180 |
|  | Thysanoptera | 0.0 ± 0.0 | 0.0 ± 0.0 | 0.0 ± 0.0 | 0.1 ± 0.1 | - | - |
|  | Larva/Immature | 2.4 ± 0.8^a^ | 7.4 ± 1.0^b^ | 5.0 ± 0.8^b^ | 13.7 ± 0.7^c^ | 23.0 | 0.001 |
| *Molluska* | |  |  |  |  |  |  |
|  | Gastropoda | 0.4 ± 0.2 | 0.2 ± 0.1 | 0.0 ± 0.0 | 0.6 ± 0.2 | 6.1^K^ | 0.107 |
| *Myriapoda* | |  |  |  |  |  |  |
|  | Chiclopoda | 0.6 ± 0.2^a^ | 0.9 ± 0.2^ab^ | 1.9± 0.6^b^ | 1.0 ± 0.2^ab^ | 4.1 | 0.032 |
|  | Diplopoda | 0.9 ± 0.3^a^ | 2.6 ± 0.1^ab^ | 3.3 ± 0.8^b^ | 1.4 ± 0.5^ab^ | 5.9 | 0.010 |
|  | **Pitfall samples** |  |  |  |  |  |  |
| *Arachnida* | |  |  |  |  |  |  |
|  | Acarina | 0.0 ± 0.0 | 0.1 ± 0.1 | 0.0 ± 0.0 | 0.1 ± 0.1 | 0.7 | 0.583 |
|  | Araneae | 0.4 ± 0.2 | 0.3 ± 0.1 | 0.1 ± 0.1 | 0.3 ± 0.1 | 1.8 | 0.198 |
|  | Opiliones | 0.3 ± 0.2 | 0.5 ± 0.2 | 0.1 ± 0.1 | 0.2 ± 0.1 | 0.6 | 0.906 |
| *Annelida* | | 0.0 ± 0.0 | 0.0 ± 0.0 | 0.0 ± 0.0 | 0.0 ± 0.0 | 2.2 | 0.542 |
| *Crustacea* | |  |  |  |  |  |  |
|  | Isopoda | 0.0 ± 0.0 | 0.0 ± 0.0 | 0.1 ± 0.1 | 0.1 ± 0.1 | 0.7 | 0.865 |
| *Entognatha* | |  |  |  |  |  |  |
|  | Collembola | 0.0 ± 0.0^a^ | 0.7 ± 0.6^b^ | 0.0 ± 0.0^a^ | 1.5 ± 0.6^b^ | 6.6 | 0.007 |
| *Insecta* | |  |  |  |  |  |  |
|  | Blattaria | 0.0 ± 0.0 | 0.2 ± 0.1 | 0.0 ± 0.0 | 0.1 ± 0.1 | 1.0 | 0.396 |
|  | Coleoptera | 0.4 ± 0.1^a^ | 0.8 ± 0.2^a^ | 0.8 ± 0.2^a^ | 3.8 ± 0.2^b^ | 37.7 | 0.001 |
|  | Dermaptera | 0.0 ± 0.0 | 0.3 ± 0.2 | 0.3 ± 0.2 | 0.1 ± 0.0 | 0.5 | 0.672 |
|  | Hemiptera | 0.1 ± 0.0^a^ | 0.2 ± 0.1^a^ | 0.1 ± 0.0^a^ | 1.1 ± 0.6^b^ | 4.3^K^ | 0.028 |
|  | Homoptera | 0.1 ± 0.1 | 0.4 ± 0.2 | 0.2 ± 0.1 | 0.0 ± 0.0 | 2.8 | 0.428 |
|  | Hymenoptera | 6.8 ± 0.5 | 8.4 ± 1.7 | 5.1 ± 0.8 | 3.9 ± 0.6 | 1.0 | 0.438 |
|  | Orthoptera | 1.1 ± 0.1 | 0.8 ± 0.2 | 1.4 ± 0.3 | 1.1 ± 0.6 | 0.2 | 0.862 |
|  | Siphonaptera | 0.0 ± 0.0 | 0.0 ± 0.0 | 0.0 ± 0.0 | 0.1 ± 0.1 | - | - |
|  | Larva/Immature | 0.5 ± 0.3^a^ | 0.5 ± 0.2^a^ | 0.4 ± 0.1^a^ | 2.2 ± 0.4^b^ | 6.4 | 0.008 |
| *Myriapoda* | |  |  |  |  |  |  |
|  | Diplopoda | 0.1 ± 0.0 | 0.0 ± 0.0 | 0.0 ± 0.0 | 0.0 ± 0.0 | - | - |
|  | *Means with the same letter are not significantly different using Tukey’s multiple-comparison procedure (p < 0.05)* | | | | | | |

| **Supp. Table 3.** Arthropod functional diversity density per m^2^ for litter samples and per trap for pitfall samples in four habitat types. Values are means ± 1 SE. Variables were compared using ANOVA, unless noted with superscript K indicating a nonparametric Kruskal-Wallis analysis | | | | | | | |
| --- | --- | --- | --- | --- | --- | --- | --- |
| Habitat type | Natural regeneration | Island | Plantation | Reference forest | F/KS test statistic | | P |
| **Litter Samples** |  | | | | | | |
| Aquatic Phytophage (herbivore) | 0.1 ± 0.1 | 0.2 ± 0.2 | 0.3 ± 0.2 | 0.5 ± 0.4 | 0.7^K^ | | 0.874 |
| Coprophage (dung consumer) | 0.1 ± 0.1 | 0.2 ± 0.1 | 0.3 ± 0.2 | 0.5 ± 0.1 | 2.1^K^ | | 0.157 |
| Saprophage (detritivore) | 4.8 ± 1.4^a^ | 12.2 ± 2.3^ab^ | 13.9 ± 2.6^b^ | 16.6 ± 2.7^b^ | 6.6 | | 0.009 |
| Mixed (Omnivore) | 5.3 ± 2.4 | 11.9 ± 1.1 | 5.8 ± 1.9 | 9.2 ± 1.3 | 3.0 | | 0.071 |
| Mycophage (fungivore) | 0.3 ± 0.3 | 0.5 ± 0.2 | 0.7 ± 0.5 | 1.8 ± 0.5 | 2.7 | | 0.092 |
| Parasite | 0.5 ± 0.3 | 1.3 ± 0.4 | 0.7 ± 0.3 | 1.6 ± 0.6 | 2.1 | | 0.160 |
| Phytophage (herbivore) | 0.5 ± 0.1^a^ | 1.4 ± 0.4a^b^ | 0.6 ± 0.3^a^ | 4.3 ± 1.8^b^ | 5.3 | | 0.015 |
| Predator (carnivore) | 5.2 ± 1.3^a^ | 12.8 ± 1.7^b^ | 9.5 ± 1.1^ab^ | 15.9 ± 1.9^b^ | 9.7 | | 0.002 |
| Seed eater (herbivore) | 0.4 ± 0.3 | 0.7 ± 0.4 | 0.4 ± 0.3 | 1.1 ± 0.6 | 0.7 | | 0.573 |
| Xylophage (wood consumer) | 0.0 ± 0.0 | 0.1 ± 0.1 | 0.0 ± 0.0 | 0.2 ± 0.1 | 4.6^K^ | | 0.202 |
| **Pitfall samples** |  | | | | | | |
| Coprophage (dung consumer) | 0.1 ± 0.1 | 0.3 ± 0.3 | 0.1 ± 0.1 | 0.1 ± 0.1 | 7.0^K^ | 0.071 | |
| Saprophage (detritivore) | 1.3 ± 0.1^a^ | 1.4 ± 0.6^a^ | 1.8 ± 0.5^ab^ | 3.9 ± 0.9^b^ | 4.3 | 0.028 | |
| Mixed (Omnivore) | 6.8 ± 0.5 | 8.4 ± 1.7 | 5.1 ± 0.8 | 3.9 ± 0.6 | 1.9 | 0.188 | |
| Mycophage (fungivore) | 0.0 ± 0.0^a^ | 0.7 ± 0.6^a^ | 0.0 ± 0.0^a^ | 1.6 ± 0.6^b^ | 11.1^K^ | 0.009 | |
| Parasite | 0.0 ± 0.0 | 0.1 ± 0.1 | 0.0 ± 0.0 | 0.1 ± 0.1 | 0.8 | 0.501 | |
| Phytophage (herbivore) | 0.3 ± 0.1 | 0.7 ± 0.2 | 0.4 ± 0.1 | 1.4 ± 0.5 | 3.1 | 0.065 | |
| Predator (carnivore) | 0.9 ± 0.3 | 1.0 ± 0.4 | 0.3 ± 0.1 | 1.2 ± 0.2 | 2.4 | 0.115 | |
| Seed eater (herbivore) | 0.0 ± 0.0 | 0.0 ± 0.0 | 0.0 ± 0.0 | 0.1 ± 0.0 | - | - | |
| Xylophage (wood consumer) | 0.0 ± 0.0 | 0.1 ± 0.0 | 0.1 ± 0.1 | 0.1 ± 0.1 | 2.0^K^ | 0.583 | |
| *Means with the same letter are not significantly different using Tukey’s multiple-comparison procedure (p < 0.05)* | | | | | | | |

| **Supp. Table 4.** PERMANOVA analysis results for litter and pitfall morphospecies and functional group data. | | | | | | | | | | |
| --- | --- | --- | --- | --- | --- | --- | --- | --- | --- | --- |
| **Litter morphospecies abundance (P values for post-hoc pair-wise comparisons)** | | | | | | | | | | |
|  |  | | Island | | | Plantation | | | | Reference forest |
|  | Natural regeneration | | 0.0265 | | | 0.0273 | | | | 0.0288 |
|  | Island | |  | | | 0.1761 | | | | 0.0300 |
|  | Plantation | |  | | |  | | | | 0.0298 |
|  | Reference forest | |  | | |  | | | |  |
| Summary of PERMANOVA (F = 2.46, P = 0.0001) | | | | | | | | | | |
|  | | | | | | | | | | |
| **Litter functional groups (P values for post-hoc pair-wise comparisons)** | | | | | | | | | | |
|  |  | | Island | | | Plantation | | | Reference forest | |
|  | Natural regeneration | | 0.0295 | | | 0.1168 | | | 0.0289 | |
|  | Island | |  | | | 0.2211 | | | 0.2037 | |
|  | Plantation | |  | | |  | | | 0.1192 | |
|  | Reference forest | |  | | |  | | |  | |
| Summary of PERMANOVA (F = 3.68, P = 0.0051) | | | | | | | | | | |
|  | | | | | | | | | | |
| **Pitfall morphospecies (P values for post-hoc pair-wise comparisons)** | | | | | | | | | | |
|  |  | Island | | Plantation | | | Reference forest | | | |
|  | Natural regeneration | 0.0857 | | 0.0579 | | | 0.0263 | | | |
|  | Island |  | | 0.1133 | | | 0.0272 | | | |
|  | Plantation |  | |  | | | 0.0316 | | | |
|  | Reference forest |  | |  | | |  | | | |
| Summary of PERMANOVA (F =3.27, P = 0.0001) | | | | | | | | | | |
|  | | | | | | | | | | |
| **Pitfall functional groups (P values for post-hoc pair-wise comparisons)** | | | | | | | | | | |
|  | | | Island | | Plantation | | | Reference forest | | |
|  | Natural regeneration | | 0.6025 | | 0.1153 | | | 0.0257 | | |
|  | Island | |  | | 0.1128 | | | 0.0279 | | |
|  | Plantation | |  | |  | | | 0.0281 | | |
|  | Reference forest | |  | |  | | |  | | |
| Summary of PERMANOVA (F = 3.98, P = 0.0009) | | | | | | | | | | |

**Suppl. Fig. 1.** Approximate arthropod sampling locations (●) in each of the four habitat types. Gray indicates areas under tree cover.


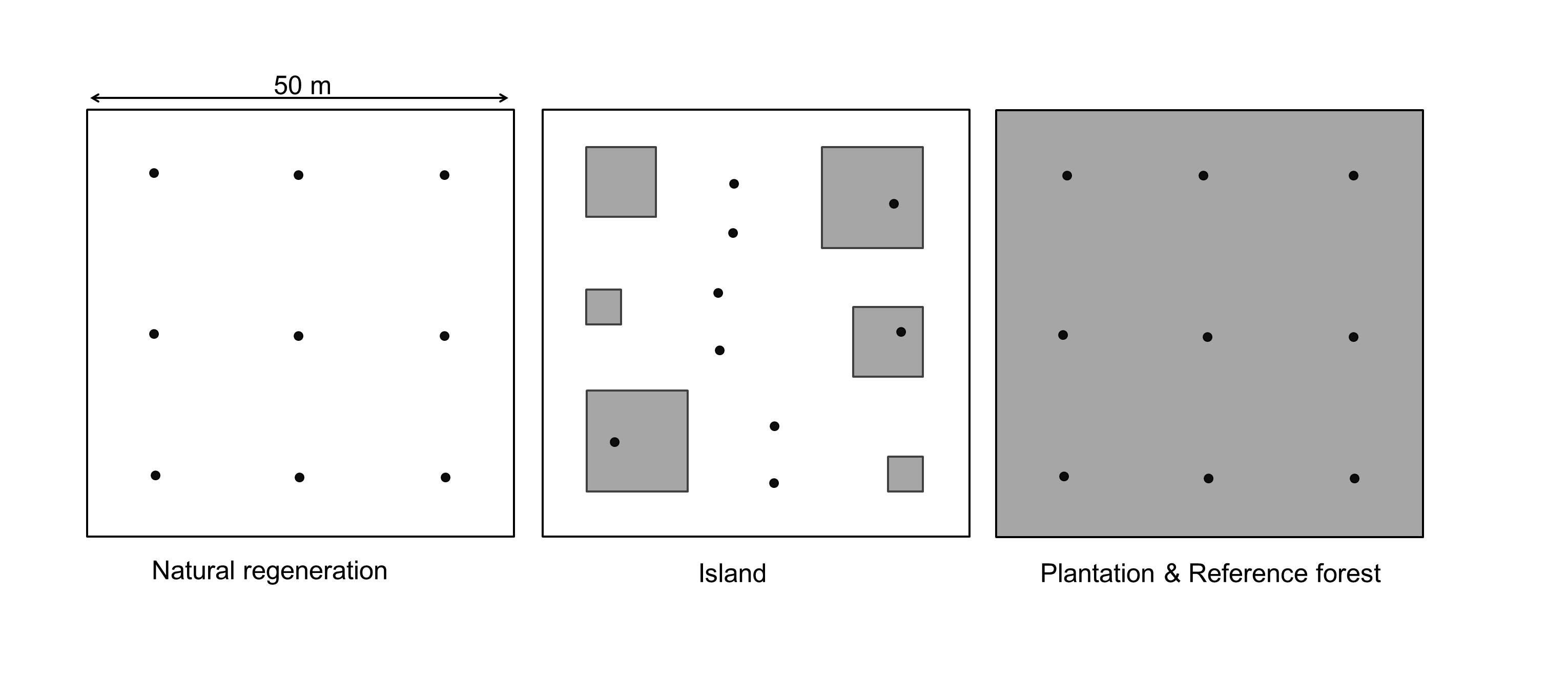

Supplement: Supplementary file 1 — Table S1. Elevation and site use history. Table S2. Abundance within orders for litter samples (individuals m−2) and pitfall samples (individuals/trap) in each habitat type (N = 4). Table S3. Arthropod functional diversity density per m2 for litter samples and per trap for pitfall samples in four habitat types. Table S4. PERMANOVA analysis results for litter and pitfall morphospecies and functional group data. Figure S1. Approximate arthropod sampling locations (●) in each of the four habitat types. [file ECE3-6-5158-s001.doc]
